# Ebola and Marburg viruses IgG detections in small ruminants and dogs from villages within outbreak areas in Gabon

**DOI:** 10.1371/journal.pone.0314801

**Published:** 2025-03-05

**Authors:** Telstar G. Ndong Mebaley, Pierre Becquart, Matthieu Fritz, Eric Elguero, Illich M. Mombo, Déborah C. Garcia, Linda Bohou Kombila, Léadisaelle H. Lenguiya, Larson Boundenga, Eric M. Leroy, Gael D. Maganga

**Affiliations:** 1 Département de Virologie, Centre Interdisciplinaire de Recherches Médicales de Franceville (CIRMF), Franceville, Gabon; 2 Institut de Recherche pour le Développement (IRD), Maladies Infectieuses et Vecteurs: Ecologie, Génétique, Evolution et Contrôle (MIVEGEC), Montpellier, France; 3 TransVIHMI, Université de Montpellier – IRD – Inserm, Montpellier, France; 4 Laboratoire National de Santé Publique, Brazzaville, République du Congo; 5 Institut National Supérieur d’Agronomie et de Biotechnologies (INSAB), Université des Sciences et Techniques de Masuku (USTM), Franceville, Gabon; University of Pécs: Pecsi Tudomanyegyetem, HUNGARY

## Abstract

The Ebola virus (EBOV) and Marburg virus (MARV) have been in circulation in Africa for several decades and are the cause of numerous outbreaks. There has been very little research on the role of domestic animals in their transmission to humans, but studies have only been conducted in dogs and pigs where relatively high levels of IgG was detected. These levels suggest that ruminants, which have not been studied, should also be investigated. This study aims at evaluating the circulation of MARV and EBOV in dogs, sheep and goats and to assess their exposure to these two viruses. Between November 2018 and March 2023, a total of 448 domestic animal sera or plasma samples, including 128 dogs, 222 goats and 98 sheep, were analyzed by serological and molecular methods. The Luminex technique was employed for the detection of IgG antibodies against EBOV NP, GP, MARV GP and VP40, while EBOV specific and pan-filovirus polymerase chain reaction amplification was used for molecular analysis. All samples tested negative for EBOV and MARV RNA. However, our results showed that 2/128 (1.5%) dogs, 1/222 (0.4%) goats and 3/98 (3.1%) sheep displayed NP and GP anti-EBOV antibodies. In addition, 2/128 (1.5%) dogs displayed GP and VP40 anti-MARV antibodies, while no antibodies were detected in goats and sheep. Over all, these results suggest that dogs and small ruminants are naturally exposed to EBOV and MARV. In the absence of clinically sick individuals, the presence of IgG-positive animals suggests various sources of exposure, such as contaminated fruits with the urine and saliva of bats or dead bats fallen on the ground ate by dogs. These contaminated substrates are both consumed by both dogs and small ruminants. The findings provide new insights into the circulation and exposure of EBOV and MARV in domestic animals, emphasising their potential use as sentinels. Furthermore, they prompt significant considerations regarding the potential risk to humans in this region.

## Introduction

Viruses in the family *Filoviridae* (commonly named filoviruses) cause deadly outbreaks of hemorrhagic fevers. Since their discovery, around 40 outbreaks have already been reported, most of them in West and Central Africa, where they have resulted in a large number of cases and deaths in both humans and non-human primates [1,2]. Filoviruses are divided into 8 genera, notably *Cuevavirus*, *Dianlovirus*, *Orthoebolavirus*, *Orthomarburgvirus*, *Oblavirus*, *Striavirus*, *Tapjovirus* and *Thamnovirus* [3], the *Orthoebolavirus* genus comprises 6 species: *Orthoebolavirus bombaliense* (Bombali virus (BOMV)), *Orthoebolavirus bundibugyoense* (Bundibugyo virus (BDBV)), *Orthoebolavirus restonense* (Reston virus (RESTV)), *Orthoebolavirus sudanense* (Sudan virus (SUDV)), *Orthoebolavirus taiense* (Tai Forest virus (TAFV)) and *Orthoebolavirus zairense* (Ebola virus (EBOV)) [3]. The genus *Orthomarburgvirus* consists of a single species known as *Orthomarburgvirus marburgense and* two distinct viruses *Marburg virus* (MARV) *and Ravn virus* (RAVV). The spillover events occurred when humans came into contact with natural hosts or secondary amplifier animals, usually through handling dead animals, mainly chimpanzees and gorillas [4,5]. Based on several results obtained from serological and molecular studies, the fruit bat *Rousettus aegyptiacus* has been described as reservoir host of MARV [6–8]. For EBOV, the reservoir host is suspected but needs to be fully characterized. Based on extensive serological studies, several fruit bats species, including *Hypsignathus monstrosus*, *Epomops franqueti*, *Myonycteris torquata*, are potential Ebola virus reservoirs [4,9,10]. However, RNA detection remains scarce, with only one study identifying it in these three frugivorous bat species [9]. Domestic animals, including pets and livestock, probably by coming into contacts with EBOV/MARV natural hosts, have been suspected to act as bridges for viral transmission between wildlife and human, mainly hunters [11]. Indeed, several serological evidence of Ebola virus infection of domestic animals (dogs and pigs) has been shown in African countries- hit by outbreaks since 2005. First, specific antibodies against EBOV have already been detected via ELISA in dogs, collected in 2004, living within the 2001- 2002 outbreak area in northeastern Gabon [12]. Seven of 79 (8.9%) samples from Libreville and Port Gentil the two largest cities, 15 of 99 (15.2%) samples from Mekambo, and 40 of 159 (25.2%) samples from villages in the Ebola outbreak area had detectable EBOV-IgG, compared to only two of 102 (2%) samples from France. Among dogs from villages with both infected animal carcasses and human cases, seroprevalence rise to 31.8% [13]. Second, in Liberia, microsphere-based multiplex immunoassay revealed that 47 of 64 (73%) dogs were potentially exposed to filoviruses. Specifically, 53.1% of the samples showed antibodies binding to EBOV-VP40, 26.6% reacted to EBOV-NP, 17.2% to EBOV-GP and 23.4% to MARV-GP [14]. The same observation was further made in Sierra Leone, where 12/300 (4%) of dogs tested were also positive by serum or plasma neutralization test, highlighting the hypothesis of natural infection of dogs by EBOV [15]. In Ghana, a country with no known Ebola Virus Disease (EVD) outbreak, 1/139 (0.7%) pig serum or plasma samples reacted against EBOV-GP [16]. In Guinea, 19/308 (6.2%) pig serum or plasma samples collected in the Conakry area sero-reacted to EBOV-NP by ELISA in 2019 [17]. The most recent study was carried out in Guinea in 2024, on 888 pig serum or plasma samples collected during 2017 and 2019, screened by ELISA and Luminex against EBOV-NP. A total of 223 (25.11%) samples were ELISA-positive and confirmed in luminex [18]. However, similar studies have never been carried out on other animals, such as small ruminants including goats and sheep, while they can be found in large numbers in every village, straying and living very closely to the villagers. In order to confirm and determine whether dogs and small ruminants (goats and sheep) are naturally exposed to EBOV and MARV during non-epidemic periods. Serological and molecular studies were carried on of these animals in villages in north-east Gabon, in a region affected by Ebola outbreaks between 2001 and 2002 and where MARV-positive bats have been frequently observed [4,19].

## Materials and methods

### Declaration of ethics

To carry out the sampling campaign, we obtained authorization to capture and collect animals from the Ministry of Water and Forests, in charge of the environment and sustainable development (Authorization No. 0247 MEFCEDD/SG/DGFAP). All applicable international and national guidelines for the care of pets were followed. The animals did not suffer, die or experience trauma. They were simply put to sleep for a limited time with ketamine and we waited for the animal to wake up each time, with the consent of the owners.

### Study areas

**Fig 1:**
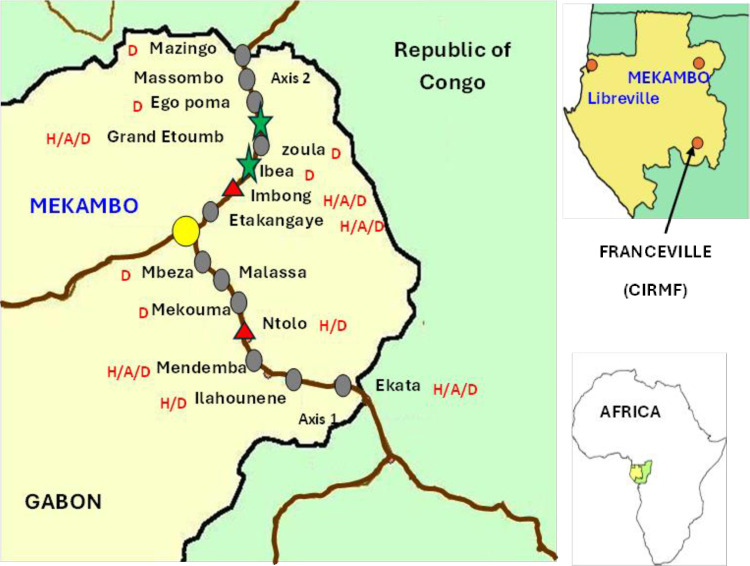
Location sources of outbreaks (2001-2002) and serology positive dogs in Gabon 2005. Reprinted from [13], under a CC BY license, with permission from Eric M. Leroy, original copyright 2024.11.21. Stars indicate the location of serology positive dogs MARV 2024. Triangles indicate the location of serology positive dogs EBOV 2024. The villages where serology dogs of EBOV in 2005 were observed are indicate by <D> . The villages where both human cases and serology dogs of EBOV in 2005 were observed are indicate by <H/D> . The villages where human cases, infected animal carcass and serology positive dogs observed are indicate by <H/A/D).

**Fig 2:**
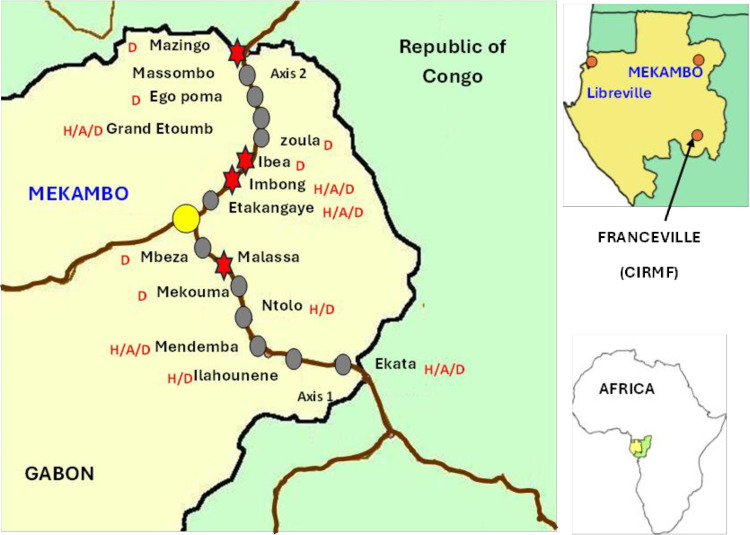
Location sources of outbreaks (2001-2002) and serology positive dogs in Gabon 2005. Reprinted from [13], under a CC BY license, with permission from Eric M. Leroy, original copyright 2024.11.21. Stars indicate the location of serology positive small ruminants EBOV 2024. The villages where serology dogs of EBOV in 2005 were observed are indicate by <D> . The villages where both human cases and serology dogs of EBOV in 2005 were observed are indicate by <H/D> . The villages where.

This study was carried out in the department of Zadié, province of Ogooué-Ivindo in northeastern Gabon, mainly comprised of primary tropical forest, in 15 villages near the town of Mekambo, capital city of the department of Zadié, where epidemics and epizootics of EVD, were confirmed between 2001 and 2002 [20]. Collections were carried in villages located along two main roads emanating from Mekambo: Mekambo-Mazingo (route #1) and Mekambo-Ekata (route #2) (Figs 1 and 2).

### Sampling and data collection

A total 448 were tested, including 153 dogs, 283 goats and 149 sheep, livestock (goats and sheep) and dogs were sampled between November 2018 and March 2023 along route #1 and route #2 (Figs 1 and 2). There is no available information on livestock population of this region. Domestic animals were selected based on the willingness of the livestock owners to participate in the study. The number of sheep and goats sampled relied on the livestock owners’ availability and their ability to restrain their animals for blood collection.

Data on species, sex, period of sampling and age (or sexual maturity stage) were collected using a standard questionnaire submitted to each animal owner. Sheep and goats were classified as young or adult according to the criterion of sexual maturity: young (under 3 years old) and adult (aged >3 years) using both morphological characters observed by the veterinarians and information provided by animal owners. All the dogs in our study were born after 2002. For each domestic animal, blood was collected in 4-ml EDTA BD vacutainer tubes (BD-Plymouth, UK) or Vacuette® dry tube (Greiner Bio-One, UK) upon jugular venipuncture and preserved in a cooler box until transport to the laboratory at the Mekambo health center. After centrifugation on site, serum or plasma was stored in liquid nitrogen before transported to CIRMF (*Centre Interdisciplinaire de Recherches Médicales de Franceville*) laboratory for storage at −80°C.

### Extraction and detection of RNA from EBOV and filoviruses

All samples were tested for the presence of the genome of the EBOV specifically and filoviruses in general, using a RT-PCR method. Briefly, RNA was extracted using the Qiamp viral RNA mini kit (Qiagen, Germany) at CIRMF’s virology laboratory and followed by a EBOV specific quantitative real-time PCR (qPCR) and by filovirus nested reverse transcription PCR (nRT-PCR). For qPCR, complementary DNA (cDNA) was first synthesized from total RNA using the High Capacity Reverse Transcription Kit (Applied Biosystems, France). The cDNA was then used with probe and primers for specific detection of EBOV using the Taqman Fast Advanced Master Mix Kit (Table 1) (Applied Biosystems, France). The filovirus nRT-PCR was conducted using two pairs of degenerate primers aimed at a 298 bp fragment of the L gene [9]. For the search of filoviruses the first round he first round was performed with the Superscript III one-step RT-PCR utilizing the Platinum Taq kit (Life Technologies, France), following the manufacturer’s guidelines. The reactions were prepared with a mixture of 10 nM of each primer (Filo A and Filo B) and 5 µL of RNA, in a final volume of 25 µL. The second round was carried out with the Platinum Taq DNA polymerase kit (Life Technologies, France), which included 10 nM of primers (Filo C and Filo D) and 2 µ L of the first-round product in a final volume of 50 µL. A positive control was also included [21].

**Table 1:** Primers and probes that are used for the detection of the EBOV and filoviruses.

Virus	Primers and probes	Sequences 5’ to 3’
EBOV	EBOZNPCONF1862	AGCTACGGCGAATACCAGAGTT
	EBOZNPCONR1943	CGTCCTCGTCTAGATCGAATAGG
	EBOZNPCONP1885	6 FAM-CTCGGAAAACGGCATGAATGCACC-BHQ1
Filoviruses	Filo A	TATMGRAATTTTTCYTTYTCATT
	Filo B	ATGTGGTGGGYTATAAWARTCACTRACAT
	Filo C	GCWAAAGCMTTYCCWAGYAAYATGATGG
	Filo D	ATAAWARTCACTRACATGCATATAACA

All serum or plasma from Gabonese animals was inactivated at 56°C for one hour and sent to MIVEGEC. (Maladies Infectieuses et Vecteurs: Ecologie, Génétique, Evolution et Contrôle) Unit at the *Institut de Recherche pour le Développement* (IRD) in Montpellier, France, for serological analysis.

### Detection of anti-EBOV and anti-MARV antibodies in domestic animals

EBOV and MARV specific IgG antibodies were detected from goats, dogs and sheep sera using a luminex-based serological test adapted to each species. For EBOV, two commercially available proteins (NP and GP) were used for the serological test as previously described [22]. Briefly, the GP was derived from the Zaire strain, Boende-Lokolia (LGC Native Antigen, United Kingdom) and the NP, recombinant Ebola virus (Zaire strain) expressed and purified from *E. coli* (LGC Native Antigen, United Kingdom). For the MARV we used the following recombinant proteins: GP from the Angolan source (IBT Bioservices, USA) and VP40 expressed and purified from *E. coli* (IBT Bioservices, USA) as in the previous study [14].

### Recombinant protein coupling to microspheres

The four proteins were covalently coupled to distincts microspheres (Magplex Microsphere, Luminex Corp, Austin, TX, USA). Briefly, 2 µg of each protein of interest were coupled to distinct MagPlex microsphere sets using the BioPlex amine coupling kit (BIO-RAD) as recommended by the manufacturer, another Magplex microspheres set were also coupled to BSA as an internal control.

#### Microsphere-based multiplex immunoassay *(MIA).*

**EBOV:** The microsphere-based multiplex immunoassay was an adapted protocol from a previous study [22]. Briefly, 50 µL of serum or plasma (1/400) diluted in assay buffer (PBS-1% BSA-0.05% Tween 20) were incubated with coupled microspheres for 30 min under agitation at 700 rpm in the dark at 27 °C. After washing, 50 µL of biotin IgG-labelled specific secondary antibody (Jackson Immuno-Research Europe Ltd., Cambridge, UK) at a 4 µg/ml in assay buffer was incubated with the microspheres for 30 minutes under agitation at 700 rpm in the dark at 27 °C. After the second wash, the microspheres were incubated for 10 min in the dark at 700 rpm with 50 µl of Streptavidin-R-Phycoerythrin (Thermo Fisher Scientific, France) at a 4 µg/ml in assay buffer. After a final wash cycle, 100 µl of assay buffer were added to the microspheres and measurements were carried out using the Luminex 200 instrument (Luminex Corp, Austin, TX, USA).

**MARV:** The microsphere-based multiplex immunoassay was an adapted protocol from a previous study [22]. Briefly, 50 µL of sera (1/400) in assay buffer (PBS-1% BSA-0.05% Tween 20) were incubated with coupled microspheres for 30 min under agitation at 700 rpm in the dark at 27 °C. After washing, 50 µL (4 µg/ml) of specific secondary antibody R-phycoerythrin (R-PE) conjugated to the rabbit anti-sheep IgG (H + L) F(ab’) fragment for sheep (Jackson Immuno-Research Europe Ltd, Cambridge, UK), for goats, the specific secondary antibody R-phycoerythrin (R-PE) conjugated to the donkey anti-goat IgG (H + L) F(ab’) fragment was used, and for dogs, the biotinylated protein A mixed with biotinylated protein G (4 μg/mL each) (Thermo Fisher Scientific, France) was used, 30 min under agitation at 700 rpm in the dark at 27 °C. After the second wash, the microspheres were incubated for 10 min in the dark at 700 rpm with 50 µl of Streptavidin-R-Phycoerythrin (Thermo Fisher Scientific, France) at a 4 µg/ml in assay buffer, step only for dogs. After a final wash cycle, 100 µl of assay buffer were added to the microspheres and measurements were carried out using the Luminex 200 instrument (Luminex Corp, Austin, TX, USA). At least 100 events were read for each bead set and binding events were displayed as median fluorescence intensities (MFI).

**Fig 3:**
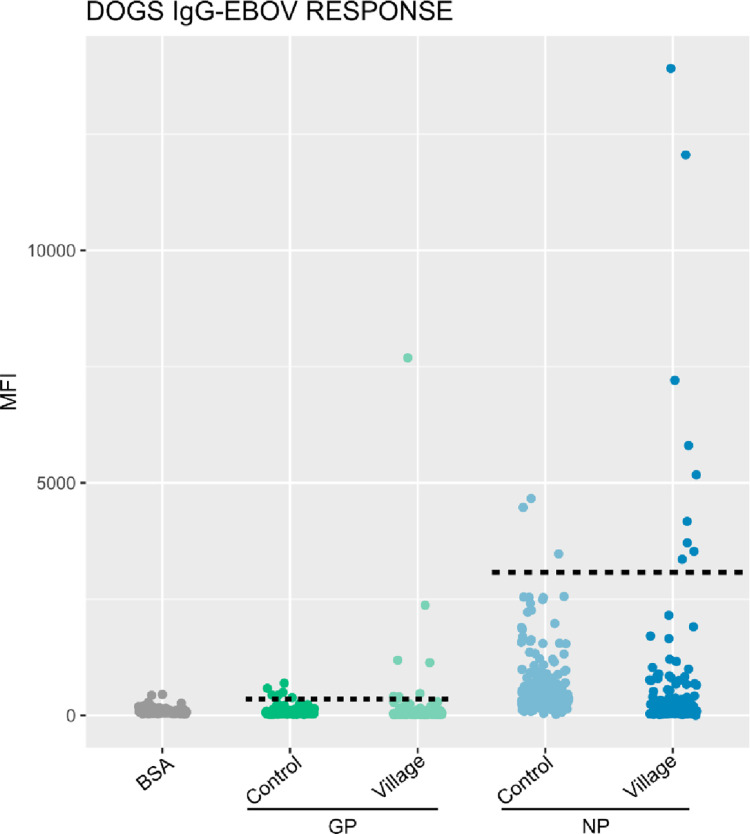
IgG responses to recombinant GP and NP proteins of the Ebola virus in dogs tested with the luminex technique. Cut-off were represented by the horizontal dotted line for each protein.

**Fig 4:**
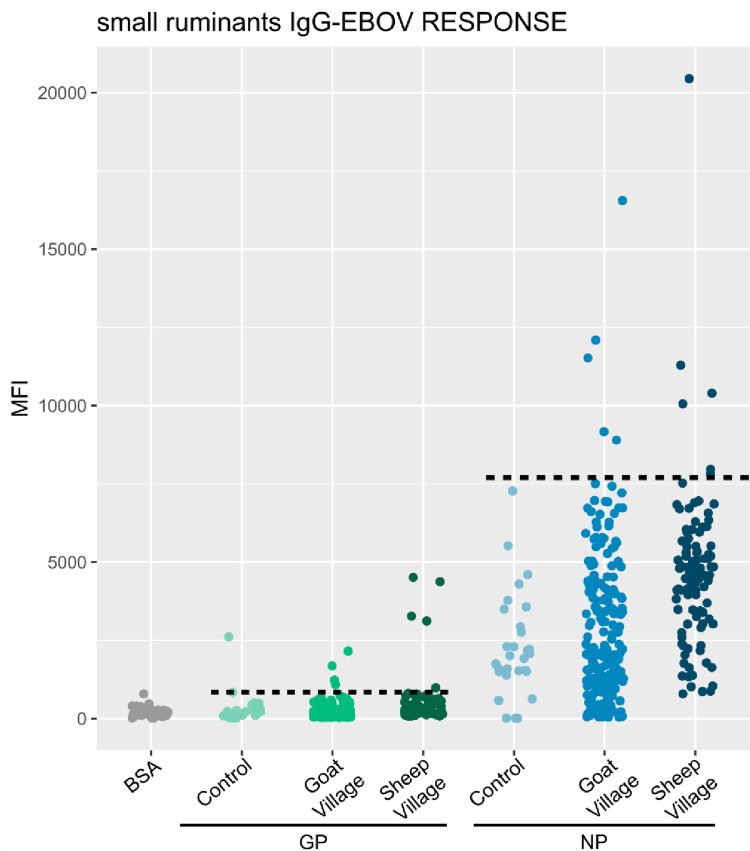
IgG responses to recombinant GP and NP proteins of the Ebola virus in small ruminants tested with the luminex technique. Cut-off were represented by the horizontal dotted line for each protein.

**Fig 5:**
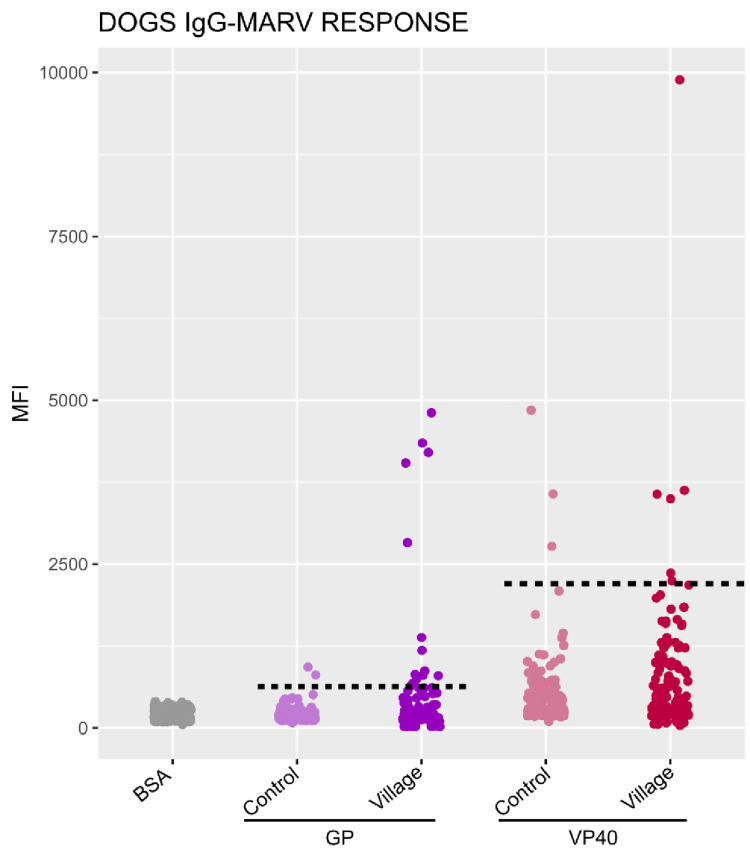
IgG responses to recombinant Marburg virus GP and VP40 proteins in dogs tested using the luminex technique. Cut-off were represented by the horizontal dotted line for each protein.

For each species studied, a panel of 200 dogs from France and 40 sheep from Libreville (an area expected to be free of EBOV and MARV epidemics) in Gabon, were used as negative controls. The positivity threshold was calculated using the following formula: the mean of the negative controls plus 3 times the standard deviation (mean +  3sd). The MIA test was considered positives when MFI was above the cut-off. The cut-off values for EBOV GP, NP proteins for were 368 and 3200, respectively, for dogs, and 850 and 7700 for small ruminants (Figs 3 and 4). For MARV, the cut-off values for GP and VP40 proteins were 650 and 2200 for dogs and 1384 and 1530 for small ruminants (Fig 5). We assessed the specificity of MIA for each protein according to EBOV and MARV. For EBOV, 7/200 dogs from the negative controls group in France had an MFI above the cut-off for GP, resulting in a specificity of 96.5%, and 4/200 dogs were positive for NP, giving a specificity of 98%. For small ruminants, the specificity was 100% specific for both proteins. For MARV, 2/200 dogs were positive for GP, giving a specificity 99%, and 1/200 dogs were positive for VP40, giving a specificity 99.5%. In small ruminants, 1/40 had an MFI above the cut-off for GP, giving a specificity of 97.5%, while all were negative for VP40. For each virus, if we apply a strict criterion to consider a sample as positive, it must be positive for both proteins tested. In other words, for EBOV, the sample must be positive for both GP and NP, and for MARV, it must be positive for both GP and VP40. Applying this criterion, all but one dogs (99,5%) from the negative control group were consider negative for both EBOV and MARV. For small ruminants, all animals from the control group (100%) were consider negative. We have therefore chosen to apply this strict positivity criterion to all our sample. The graphs presented were produced using R software version 4.2.1.

## Results

### Detection of EBOV by RT-PCR and qPCR and antibodies detection in domestic animals

All 448 samples were tested negative for EBOV and filoviruses by RT-PCR and qPCR.

A total of 448 sera (128 dogs, 222 goats and 98 sheep) from domestic animals were analyzed using the luminex technology for the detection of IgG specifically targeting the GP and NP proteins for the EBOV and GP and VP40 for the MARV. Regarding anti-EBOV IgG, 2 out of 128 dogs (1.5%) and 4 out of 320 small ruminants (1.9%) were positive; specifically, 1 out of 222 goats (0.4%) and 3 out of 98 sheep (3.1%) tested positive (Table 2). For anti-MARV IgG, 2 out of 128 dogs (1.5%) were positive, while no small ruminants showed positive results (Table 2). The positive animals came not only from former EVD outbreaks (Ntolo, Imbong and Grand Etoumbi) (Figs 1 and 2), but also from villages where no cases of Ebola had been observed during epidemics that occurred in Gabon (Mazingo, Ibéa and Malassa) (Figs 1 and 2).

**Table 2:** Serological status (IgG) of MARV and EBOV in domestic animals sampled in the Zadié department between 2018 and 2023.

Species	Tested	Positive EBOV with two proteins (%)	Positive EBOV with one protein (%)	Positive MARV with two proteins (%)	Positive MARV with one protein (%)
Dogs	128	2 (1.5)	17 (13.2)	2 (1.5)	19 (14.8)
Goats	222	1 (0.4)	5 (2.2)	0	0
Sheep	98	3 (3.1)	20 (20.4)	0	0
Total	448	6 (1.3)	42 (9.3)	2 (0.4)	19 (4.2)

## Discussion

Unlike previous studies, which were conducted soon after an outbreak with a high probability of close contact between domestic animals and infected humans or animal carcasses, our study takes place to between 16 and 21 years after the last known epidemic in Gabon. Moreover, we tested for serological and molecular markers in several domestic animals that had never been in contact with patients from the last 2001-2002 outbreak.

Serological testing revealed a seroprevalence of 2/128 (1.5%) for EBOV-IgG and 2/128 (1.5%) for MARV-IgG and 4/320 (1.9%) for EBOV-IgG in dogs and small ruminants respectively living both in the 2001 to 2002 Ebola outbreak area and in the area where several bats were found PCR positive for MARV [9,10,19] (Table 2). The desired positivity criteria (2 positive proteins) greatly reduced the likelihood of cross-reactivity. These seroprevalences are lower than those found in several other studies conducted in Gabon, Liberia, Ghana and Guinea, which were carried out less than three years after Ebola outbreaks [12–15]. The difference between the results of these studies and ours can be explained by the length of time between the last epidemic and the sampling of animals from the villages. The prevalence observed in the other studies mainly included animals that had potentially been in contact with patients throughout the outbreak periods, which probably explains the high exposure rates recorded in these studies. In contrast, our study was conducted between 16 and 21 years after the last epidemic, which de facto excludes animals exposed to the virus with sick persons during the epidemic. In fact, the dogs in our study are not the same as those that lived during the last EBOV epidemic or those involved in the Alléla study conducted in 2005 [13], The same applies to small ruminants, which have a life expectancy of no more than 12 years. Additionally, in these regions far from large cities, small ruminants are primarily utilized for livestock purposes, particularly for sale during weddings and burial ceremonies or for the consumption of animal protein. This practice further reduces their presence in these villages compared to their average lifespan. Therefore, identifying positive small ruminants is equivalent to demonstrating recent exposure to the reservoir(s) or intermediary(s) of EBOV or MARV. For small ruminants (goats and sheep), the primary hypothesis for this natural exposition is likely related to fruits contaminated by bat saliva. Fruit bats, from species suspected to be EBOV reservoirs, are abundant in the villages where the sampling was conducted. These bats roost in large numbers on trees and consume fruits, especially within and around villages. As a result, partially eaten fruits (such as mangoes and safou), along with fiber and seed remnants, are often found on the ground beneath the trees. Goats and sheep, which graze freely in these villages, consume the fruit remnants they find on the ground. Thus, it is possible that EBOV antigenic stimulation or aborted infection could occur when these animals eat fruits contaminated with bat saliva, which may contain infectious virus, or inactivated virus. Our hypotheses are supported by several studies that have demonstrated the presence of viruses in the saliva or salivary gland of wild-caught or experimentally infected bats, in particular MARV, rabies virus and Rio Bravo virus (RBV) [23–25]. Moreover, a recent study has demonstrated the persistence of MARV in eaten fruit discarded by experimentally infected Egyptian bats [26]. In addition to this last hypothesis, dogs may have been exposed to the virus when eating dead animals (bats or intermediary animal species such as great apes) found on the ground in villages or in the forest during hunting. They could also have been expose within or near villages, indeed it is known that they eat small dead animals found near villages, as well as the internal organs of wild animals hunted and slaughtered by villagers [27]. Finally, the least likely hypothesis, but one that should not be ruled out, is based on potential direct or indirect exposure to asymptomatic or paucisymptomatic human who, for reasons as yet unknown, may not develop any alarming symptoms but may have shed the virus through secretions such as saliva, sweat or urine [28,29]. Although our positivity criteria favor a significant reduction in cross-reactions, it should be noted that we that we cannot definitively state that there is no possibility of cross-reactions. Furthermore, in the absence of genetic material, or other confirmation techniques such as seroneutralisation, we cannot exclude the circulation of a virus closely related to EBOV or MARV in this region.

Our study highlights the value of assessing exposure in domestic animals with different dietary and behavioral habits than dogs. This approach could help identify potential routes of virus exposure. In future studies, it would be interesting to investigate exposure through contaminated fruit. Finally, this study suggests that small ruminants could also serve as sentinels for monitoring the circulation of these viruses in a region where dog access is difficult or poses a health risk. Although we cannot exclude the possibility of an unknown filovirus circulating, these results collectively suggest that, nearly 20 years after the last human epidemic in this region, EBOV and MARV are still present in the region’s wildlife. This study underscores the importance of better understanding the natural cycle of filoviruses to more effectively anticipate, prevent, and combat future outbreaks.

## References

[1] Feldmann H, Geisbert TW. Ebola haemorrhagic fever. Lancet. 2011;377(9768):849–62. doi: 10.1016/S0140-6736(10)60667-8 21084112 PMC3406178

[2] Leroy ÉM, Maganga GD. Diversité des modalités de transmission du virus Ébola à l’homme. Bulletin de l’Académie Vétérinaire de France. 2018;171(2):128–36. doi: 10.4267/2042/69030

[3] Biedenkopf N, Bukreyev A, Chandran K, Di Paola N, Formenty PBH, Griffiths A, et al. Renaming of genera Ebolavirus and Marburgvirus to Orthoebolavirus and Orthomarburgvirus, respectively, and introduction of binomial species names within family Filoviridae. Arch Virol. 2023;168(8):220. doi: 10.1007/s00705-023-05834-2 37537381

[4] Pourrut X, Kumulungui B, Wittmann T, Moussavou G, Délicat A, Yaba P, et al. The natural history of Ebola virus in Africa. Microbes Infect. 2005;7(7–8):1005–14. doi: 10.1016/j.micinf.2005.04.006 16002313

[5] Leroy EM, Rouquet P, Formenty P, Souquière S, Kilbourne A, Froment J-M, et al. Multiple Ebola virus transmission events and rapid decline of central African wildlife. Science. 2004;303(5656):387–90. doi: 10.1126/science.1092528 14726594

[6] Towner JS, Amman BR, Sealy TK, Carroll SAR, Comer JA, Kemp A, et al. Isolation of genetically diverse Marburg viruses from Egyptian fruit bats. PLoS Pathog. 2009;5(7):e1000536. doi: 10.1371/journal.ppat.1000536 19649327 PMC2713404

[7] Amman BR, Swanepoel R, Nichol ST, Towner JS. Ecology of filoviruses. In: Marburg-and Ebolaviruses: From Ecosystems to Molecules. 2017. p. 23–61. 10.1007/82_2017_10 28710694

[8] Amman BR, Carroll SA, Reed ZD, Sealy TK, Balinandi S, Swanepoel R, et al. Seasonal pulses of Marburg virus circulation in juvenile *Rousettus aegyptiacus* bats coincide with periods of increased risk of human infection. 2012. 10.1371/journal.ppat.1002877 PMC3464226 23055920

[9] Leroy EM, Kumulungui B, Pourrut X, Rouquet P, Hassanin A, Yaba P, et al. Fruit bats as reservoirs of Ebola virus. Nature. 2005;438(7068):575–6. doi: 10.1038/438575a 16319873

[10] Pourrut X, Souris M, Towner JS, Rollin PE, Nichol ST, Gonzalez J-P, et al. Large serological survey showing cocirculation of Ebola and Marburg viruses in Gabonese bat populations, and a high seroprevalence of both viruses in *Rousettus aegyptiacus*. BMC Infect Dis. 2009;9:159. doi: 10.1186/1471-2334-9-159 19785757 PMC2761397

[11] Kock RA, Begovoeva M, Ansumana R, Suluku R. Searching for the source of Ebola: the elusive factors driving its spillover into humans during the West African outbreak of 2013-2016. Rev Sci Tech. 2019;38(1):113–22. doi: 10.20506/rst.38.1.2946 31564736

[12] Leroy EM, Souquière S, Rouquet P, Drevet D. Re-emergence of Ebola haemorrhagic fever in Gabon. The Lancet. 2002;359(9307):712. doi: 10.1016/s0140-6736(02)07796-6 11879899

[13] Allela L, Boury O, Pouillot R, Délicat A, Yaba P, Kumulungui B, et al. Ebola virus antibody prevalence in dogs and human risk. Emerg Infect Dis. 2005;11(3):385–90. doi: 10.3201/eid1103.040981 15757552 PMC3298261

[14] Haun BK, Kamara V, Dweh AS, Garalde-Machida K, Forkay SSE, Takaaze M, et al. Serological evidence of Ebola virus exposure in dogs from affected communities in Liberia: A preliminary report. PLoS Negl Trop Dis. 2019;13(7):e0007614. doi: 10.1371/journal.pntd.0007614 31329600 PMC6684096

[15] Fischer K, Suluku R, Fehling SK, Jabaty J, Koroma B, Strecker T, et al. Ebola virus neutralizing antibodies in dogs from sierra Leone, 2017. Emerg Infect Dis. 2020;26(4):760–3. doi: 10.3201/eid2604.190802 32186496 PMC7101121

[16] Ogawa H, Ohya K, Ayizanga R, Miyamoto H, Shigeno A, Yamada M, et al. Detection of anti-ebolavirus antibodies in Ghanaian pigs. J Vet Med Sci. 2022;84(11):1491–4. doi: 10.1292/jvms.22-0186 36123040 PMC9705824

[17] Fischer K, Jabaty J, Suluku R, Strecker T, Groseth A, Fehling SK, et al. Serological Evidence for the Circulation of Ebolaviruses in Pigs From Sierra Leone. Journal Infect Dis. 2018;218(suppl_5):S305–11. doi: 10.1093/infdis/jiy330 29982580

[18] Grayo S, Camara A, Doukouré B, Ellis I, Troupin C, Fischer K, et al. Geographic disparities in domestic pig population exposure to Ebola viruses, Guinea, 2017-2019. Emerg Infect Dis. 2024;30(4):681–90. doi: 10.3201/eid3004.231034 38526081 PMC10977825

[19] Maganga GD, Bourgarel M, Ebang Ella G, Drexler JF, Gonzalez J-P, Drosten C, et al. Is Marburg virus enzootic in Gabon? J Infect Dis. 2011;204(Suppl 3)S800-3. doi: 10.1093/infdis/jir358 21987754

[20] Nkoghe D, Formenty P, Leroy E, Nnegue S, Edou SO, Ba JI, et al. Multiple Ebola virus haemorrhagic fever outbreaks in Gabon, from October 2001 to April 2002. Bull Soc Pathol Exot. 2005;98(3):224–9. 16267965

[21] Rouquet P, Froment J-M, Bermejo M, Kilbourn A, Karesh W, Reed P, et al. Wild animal mortality monitoring and human Ebola outbreaks, Gabon and Republic of Congo, 2001-2003. Emerg Infect Dis. 2005;11(2):283–90. doi: 10.3201/eid1102.040533 15752448 PMC3320460

[22] Mombo IM, Fritz M, Becquart P, Liegeois F, Elguero E, Boundenga L, et al. Detection of Ebola virus antibodies in fecal samples of great apes in Gabon. Viruses. 2020;12(12):1347. doi: 10.3390/v12121347 33255243 PMC7761173

[23] Constantine DG, Woodall DF. Latent infection of Rio bravo virus in salivary glands of bats. Public Health Rep (1896). 1964;79(12):1033–9. doi: 10.2307/4592318 14234343 PMC1915554

[24] Amman BR, Jones ME, Sealy TK, Uebelhoer LS, Schuh AJ, Bird BH, et al. Oral shedding of Marburg virus in experimentally infected Egyptian fruit bats (*Rousettus aegyptiacus*). J Wildlife Dis. 2015;51(1):113–24. doi: 10.7589/2014-08-198 25375951 PMC5022530

[25] Abir MH, Rahman T, Das A, Etu SN, Nafiz IH, Rakib A, et al. Pathogenicity and virulence of Marburg virus. Virulence. 2022;13(1):609–33. doi: 10.1080/21505594.2022.2054760 35363588 PMC8986239

[26] Amman BR, Schuh AJ, Albariño CG, Towner JS. Marburg virus persistence on fruit as a plausible route of bat to primate filovirus transmission. Viruses. 2021;13(12):2394. doi: 10.3390/v13122394 34960663 PMC8708721

[27] Hambese H, Hadush T, Tilahun A, Teshale A, Getachew A. Ebola virus disease in domestic and wild animals: a review. J Pharm Alternat Med. 2016;10:54.

[28] Leroy EM, Epelboin A, Mondonge V, Pourrut X, Gonzalez JP, Muyembe-Tamfum JJ, et al. Human Ebola outbreak resulting from direct exposure to fruit bats in Luebo, Democratic Republic of Congo, 2007. Vector Borne Zoonotic Dis. 2009;9(6):723–8. doi: 10.1089/vbz.2008.0167 19323614

[29] Bausch DG, Towner JS, Dowell SF, Kaducu F, Lukwiya M, Sanchez A, et al. Assessment of the risk of Ebola virus transmission from bodily fluids and fomites. Journal Infect Dis. 2007;196(Suppl 2)S142–7. doi: 10.1086/520545 17940942

